# A new, alpine species of *Lissodesmus* Chamberlin, 1920 from Tasmania, Australia (Diplopoda, Polydesmida, Dalodesmidae)

**DOI:** 10.3897/zookeys.754.25704

**Published:** 2018-05-03

**Authors:** Robert Mesibov

**Affiliations:** 1 West Ulverstone, Tasmania 7315, Australia

**Keywords:** Diplopoda, Polydesmida, Dalodesmidae, Tasmania, Australia

## Abstract

*Lissodesmus nivalis* **sp. n.** is described from 1450–1550 m elevation on the treeless, alpine Ben Lomond plateau in northeast Tasmania, Australia. The new species is distinguished from all other Tasmanian and Victorian *Lissodesmus* species by a unique combination of gonopod telopodite features: solenomere without a pre-apical process, tibiotarsus Y-shaped, femoral process L-shaped with forked tips, prefemoral process with a long comb of teeth below an irregularly dentate apical margin, and a roughened “shoulder process” near the base of the prefemoral process.

## Introduction

**Figure 1.**
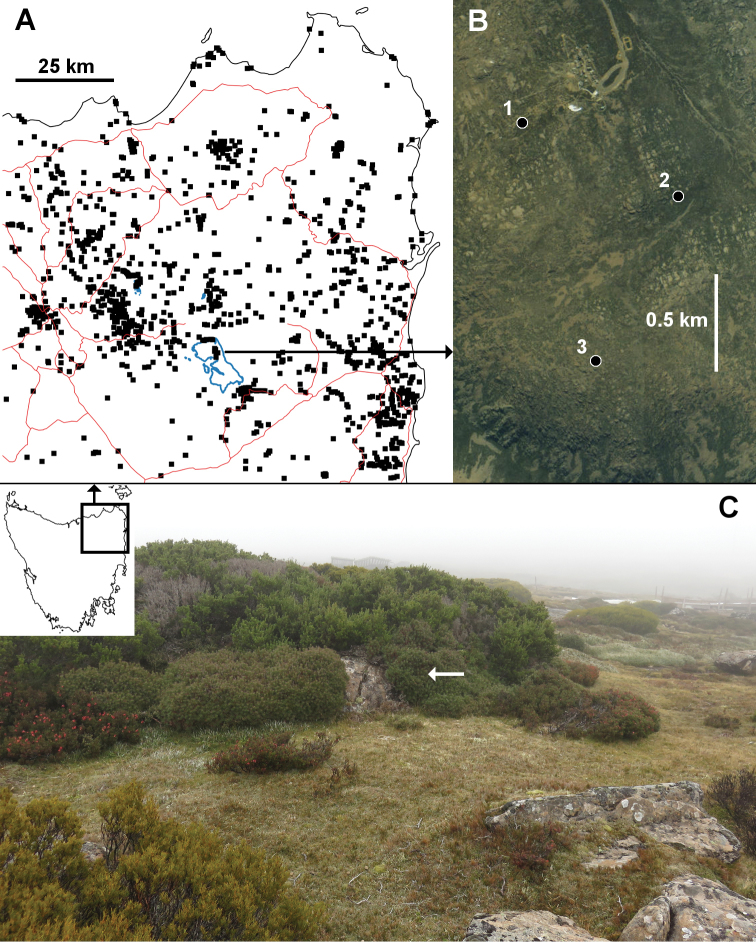
**A** Northeast Tasmania with millipede sampling sites, 1934–2018 (black squares), major roads (thin red lines) and 1300 m elevation contours (thick blue lines); the large, rectangular block above 1300 m is the Ben Lomond plateau **B** Aerial photograph of part of the Ben Lomond plateau with *Lissodesmus nivalis* sp. n. localities: 1 = Ben Lomond ski village (type locality), 2 = Surprise Vale, 3 = Giblin Fells **C** Ben Lomond ski village collecting site (1 in **B**), 2 April 2018; white arrow indicates the rock-hugging shrub beneath which the holotype and paratypes of *L. nivalis* sp. n. were found. Sampling sites in **A** from Mesibov (2006–2018) for named species, and the author’s unpublished records for undescribed species. Image in **B** from https://maps.thelist.tas.gov.au/listmap/app/list/map. Rectangle in inset map in **C** shows extent of map **A** both maps are Mercator projections.

Several species of the millipede family Dalodesmidae can be found in treeless alpine areas of Tasmania, among them *Dasystigma margaretae* (Jeekel, 1984), which was collected on an alpine “cushion plant” at 1150 m at the type locality on Tasmania’s Central Plateau (Mesibov 2003). However, until recently the only Tasmanian dalodesmid known exclusively from above the treeline was *Noteremus summus* Mesibov, 2009 from the summit of Mt Weld (1100–1300 m) in the south of the island (Mesibov 2009). The new species described here is so far known only from ca 1450–1550 m on the Ben Lomond plateau in northeast Tasmania (Fig. 1). Its discovery in 2017 was remarkable for another reason: northeast Tasmania has been intensively sampled for millipedes by the author and other collectors over many years (Fig. 1A). I therefore thought the list of the region’s dalodesmid fauna might be complete, apart from very small, inconspicuous and geographically restricted forms yet to be collected. Unexpectedly, the new Ben Lomond species is a large and conspicuous addition to the Tasmanian and Victorian genus *Lissodesmus* Chamberlin, 1920, which now includes 30 species (Mesibov 2006–2018).

## Materials and methods

All specimens are stored in 80% ethanol in the Queen Victoria Museum and Art Gallery (QVM). Several legs of the holotype were removed and placed in 95% ethanol before the rest of the specimen was preserved. Freshly collected specimens were examined and measured using a Nikon SMZ800 binocular dissecting microscope, and stacks of colour images were manually generated using a Canon EOS 1000D digital SLR camera mounted on the Nikon SMZ800 fitted with a beam splitter. Images were then focus-stacked with Zerene Stacker 1.04 software. The gonopods and one leg 7 of the male paratype were cleared in 80% lactic acid and temporarily mounted in a 1:1 glycerol:water mixture for examination. The gonopods were imaged using an eyepiece video camera mounted on an Amscope binocular microscope. Preliminary drawings were traced from printed copies of the images, then corrected by reference to the actual gonopod. Figures were composed using GIMP 2.8 and maps with QGIS 2.14.

Locality details are given with latitude and longitude in decimal degrees based on the WGS84 datum. The estimated uncertainty for a locality is the radius of a circle around the given position in metres.

Terminology of gonopod telopodite parts as in Mesibov (2006).

Abbreviation: QVM = Queen Victoria Museum and Art Gallery, Launceston, Tasmania, Australia.

## Results

### Order Polydesmida Pocock, 1887

#### Suborder Dalodesmidea Hoffman, 1980

##### Family Dalodesmidae Cook, 1896

###### Genus Lissodesmus

Taxon classificationAnimaliaPolydesmidaDalodesmidae

Chamberlin, 1920

Lissodesmus : Chamberlin 1920: 135; Attems 1940: 490; Jeekel 1971: 336; Hoffman 1980: 185; Jeekel 1982: 12; 1983: 146, 150; 1984: 85, 89; 1985: 50, 51; Mesibov 2003: 198; 2004: 21; 2006: 108; 2008: 2, 49.
Australopeltis : Johns 1964: 47 (as subgenus of Pseudoprionopeltis Carl, 1902); Hoffman 1980: 184 (raised to genus); Jeekel 1982: 12; 1983: 150 (synonymised with Lissodesmus); Shelley et al. 2000: 86; Mesibov 2006: 108; 2008: 49. (Type species Pseudoprionopeltis martini Carl, 1902, by original designation.)

####### Type species.

*Lissodesmus modestus* Chamberlin, 1920, by original designation.

####### Other assigned species.

*Lissodesmus adrianae* Jeekel, 1984, *L. alisonae* Jeekel, 1984, *L. anas* Mesibov, 2006, *L. bashfordi* Mesibov, 2006, *L. blackwoodensis* Mesibov, 2006, *L. catrionae* Mesibov, 2006, *L. clivulus* Mesibov, 2006, *L. cognatus* Mesibov, 2006, *L. cornutus* Mesibov, 2006, *L. devexus* Mesibov, 2006, *L. dignomontis* Mesibov, 2006, *L. gippslandicus* Mesibov, 2006, *L. grampianensis* Mesibov, 2008, *L. hamatus* Mesibov, 2006, *L. horridomontis* Mesibov, 2006, *L. inopinatus* Mesibov, 2006, *L. johnsi* Mesibov, 2006, *L. latus* Mesibov, 2006, *L. macedonensis* Mesibov, 2006, *L. martini* (Carl, 1902), *L. milledgei* Mesibov, 2006, *L. montanus* Mesibov, 2006, *L. nivalis* sp. n., *L. orarius* Mesibov, 2006, *L. otwayensis* Mesibov, 2006, *L. peninsulensis* Mesibov, 2006, *L. perporosus* Jeekel, 1984, *L. plomleyi* Mesibov, 2006, *L. tarrabulga* Mesibov, 2006.

###### Lissodesmus nivalis sp. n.

Taxon classificationAnimaliaPolydesmidaDalodesmidae

http://zoobank.org/688C85B2-1C5F-4ACE-AA6C-6C663BF25788

Figs 2 , 3

####### Holotype.

Male, Ben Lomond ski village, Tasmania, -41.5357, 147.6618, ±25 m, 1490 m a.s.l., 2 April 2018, K. Bonham and R. Mesibov, QVM 2018:23:0038.

####### Paratypes.

1 male, 1 female, details as for holotype, QVM 2018:23:0039.

####### Other material

. Tasmania, QVM: 1 male, Giblin Fells, Ben Lomond, -41.5471, 147.6666, ±100 m, 1540 m a.s.l., 15 April 2017, K. Bonham, 2017:23:0185, gonopod telopodite with femoral processes broken off; 1 stadium VI male, Surprise Vale, Ben Lomond, -41.5392, 147.6719, ±50 m, 1450 m a.s.l., 20 November 2017, R. Mesibov, 2017:23:0244.

####### Diagnosis.

Distinguished from all other *Lissodesmus* species by a unique combination of character states of gonopod telopodite processes: solenomere without pre-apical process, tibiotarsus Y-shaped, femoral process L-shaped with forked tips, prefemoral process with long comb of teeth below irregularly dentate apical margin, roughened “shoulder process” near base of prefemoral process.

####### Description.

Male/female approximate measurements: length 25/25 mm, midbody vertical diameter 2.1/2.5 mm, midbody width across paranota 3.2/3.5 mm. Live specimens yellowish-brown to chestnut brown with pinkish red antennae (darker distally) and pinkish red legs (darker basally); rings darkest at posterior metatergal margin and along paranotal margins.

**Figure 2.**
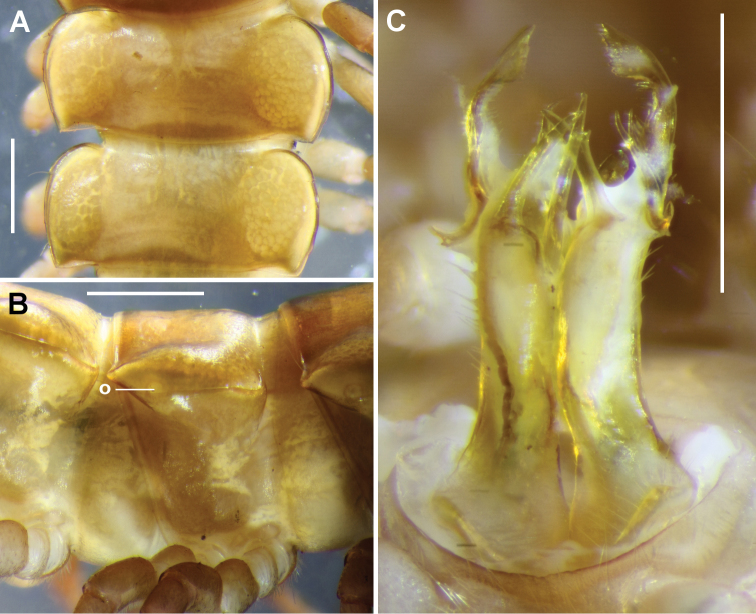
*Lissodesmus nivalis* sp. n., holotype male five days after killing and preserving, QVM 2018:23:0038. **A** Dorsal view of rings 10 and 11 **B** Right lateral view of rings 9–11; **o** = ozopore **C** Posterior view of gonopods in situ. Scale bars: 1.0 mm.

Male with vertex sparsely setose and frons moderately so. Antennal sockets separated by 2X socket diameter. Antenna short, just reaching ring 3 when manipulated backwards; relative length of antennomeres 2>(3,6)>(4,5), antennomere 6 widest. Collum slightly narrower than head, slightly wider than tergite 2; anteriorly very slightly convex, laterally slightly convex; posterior edge medially a little emarginate; corners rounded. Tergite width increasing gradually from rings 2–6, then subequal, then decreasing 17–19. Waist (Fig. 2A, B) well-defined, with faint longitudinal striations. Prozonites and metazonites with faint cellular sculpturing; limbus composed of close-set flat tabs. Paranota (Fig. 2A, B) smooth, swollen, wide (ratio of overall width to prozonite width ca 1.4 on midbody ring); anterior shoulder tightly curved, projected anteriorly; lateral margin slightly convex, sometimes with small notches detectable, the first and last usually with 1 short seta on anterior corner of notch; posterior corner rounded, not extending past posterior metatergite margin on most rings, produced as rounded tooth on rings 17–19; posterior corner seta prominent, erect. Ozopore (Fig. 2B) small, opening dorsolaterally close to paranotal margin and anterior to posterior paranotal corner; pore formula 5, 7, 9, 10, 12, 13, 15–19. Spiracles with rims slightly raised above pleural surface; anterior spiracle on diplosegments larger, posterior spiracle about midway between leg bases. Sternites about as long as wide, with fine, short, sparse setae; transverse impression much deeper than longitudinal. Legs short; from legpair 3 prefemur greatly swollen dorsally, femur somewhat swollen dorsally, gradually less so posteriorly; most legs with postfemur and tibia expanded ventrodistally; relative podomere lengths tarsus>femur>prefemur>(postfemur, tibia) on midbody legs; tarsus straight. From legpair 3, sphaerotrichomes on prefemur, femur, postfemur, tibia and tarsus, densest on tibia and tarsus; each sphaerotrichome hemispherical with blunt-tipped, tapered setal shaft inclined distoventrally; dense brush setae on prefemur and femur, tapering with blunt tips; no sphaerotrichomes or brush setae on last 2 legpairs. Pre-anal ring with sparse, long setae; hypoproct paraboloid; epiproct extending a little past anal valves, sides slightly emarginate, tip truncate between 2 short, rounded, apical bumps; spinnerets in square array in shallow, low-walled cavity just ventral to epiproct tip.

Gonopore small, opening on rounded, mediodistal enlargement of leg 2 coxa. Gonopod telopodites extending to leg 5 bases when retracted; bases of legs 6 and 7 well-separated, coxae of legs 6 and 7 slightly swollen ventrally; sternite between legs 6 and 7 slightly excavate, with brushes of long, stiff setae on sternite just medial to coxae. Aperture ovoid but with anterior margin straight, wider than long, about 1/2 the width of ring 7 prozonite, rim slightly raised laterally.

**Figure 3.**
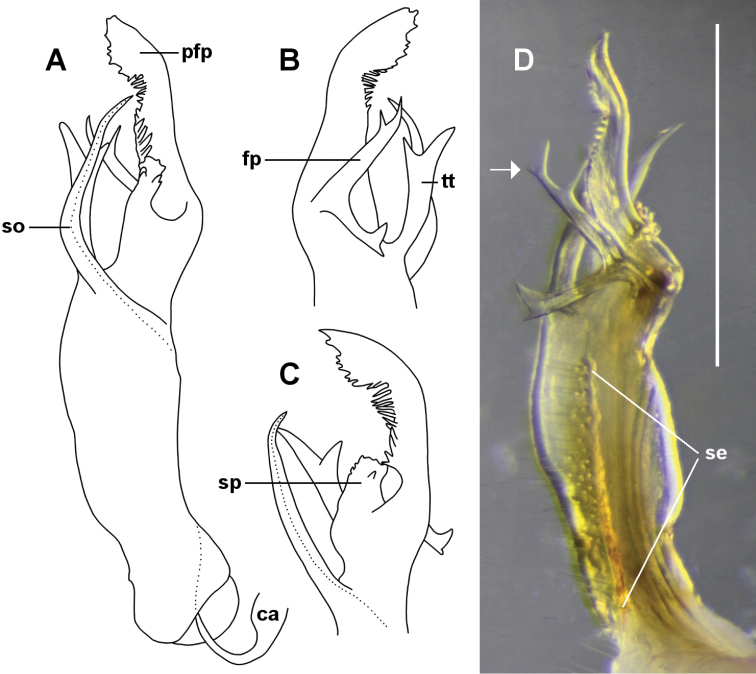
*Lissodesmus nivalis* sp. n., gonopod telopodites of paratype male ex QVM 2018:23:0039. Medial (**A**), posterolateral (**B**) and anteromedial (**C**) views of right telopodite; drawings not to scale and setation not shown **D** Lateral view of left telopodite; scale bar = 1.0 mm. **fp** = femoral process, **pfp** = prefemoral process, **se** = band of setae on posterolateral surface, **so** = solenomere, **sp** = “shoulder process”, **tt** = tibiotarsus. Arrow in **D** indicates unusually forked tip of **fp**; dotted lines in **A** and **C** indicate course of prostatic groove.

Gonopods: gonocoxae truncate-conical, tapering distally, lightly joined medially, moderately setose on posterobasal surface. Telopodite (Figs 2C, 3) long, slender, gently curving anteriorly from base, with a band of long setae (Fig. 3C) on posterolateral surface from telopodite base almost to level of solenomere base. Telopodite base tapering from thickened basal rim. Cannula prominent, entering telopodite base in excavation lined with fine setae; prostatic groove running on anterior surface of telopodite before joining base of solenomere, opening at solenomere tip. Solenomere thin, rod-like, tapering to point, arising at ca 1/2 telopodite height on anteromedial surface, directed a little posterodistally before curving anterodistally. Tibiotarsus arising on posterior surface a little distal to level of solenomere base, thicker than solenomere, directed distomedially, apex bifurcated, the tips blunt. Femoral process arising on anterolateral surface distal to tibiotarsus origin, L-shaped; the longer portion of the “L” directed posterodistally with a forked tip; the shorter portion of the “L” directed posterobasally, also with forked tip but with distal portion of fork larger than basal portion. In distal 1/3 of telopodite, prefemoral process separated on posteromedial side from prominent, tab-like “shoulder” process with irregularly and bluntly toothed margin. Prefemoral process flattened anterolaterally, curving medially near obliquely truncate apex, with apical margin irregularly and bluntly toothed and with ca 15 discrete teeth forming comb on posterior margin.

Female closely resembling male but a little wider; legs not swollen. Genital aperture with posterior margin rounded-triangular medially; cyphopods not examined.

####### Name.

Latin *nivalis*, of snow; adjective. This species spends several months each winter with its habitat covered in snow.

####### Distribution.

So far known only from alpine moorland and shrubland at three localities on the Ben Lomond plateau in northeast Tasmania (Fig. 1A, B). Found in peaty material under prostrate and rock-hugging alpine shrubs (Fig. 1C).

####### Remarks.

The gonopod telopodite of *L. nivalis* sp. n. shares several features with other Tasmanian *Lissodesmus* species. As in *L. anas* and *L. horridomontis*, for example, the prefemoral process is offset laterally by the distal development of a roughened, tab-like “shoulder” process. The tibiotarsus has a bifurcated tip, as in *L. cornutus* and *L. montanus*, and the femoral process is L-shaped, as in *L. clivulus* and *L. latus*. However, the distinctive combination of telopodite characters in *L. nivalis* sp. n. makes it hard to judge from morphology what its nearest relation in the genus might be. It is quite unlike the five other *Lissodesmus* species found in the Ben Lomond area, namely *L. adrianae*, *L. cognatus*, *L. devexus*, *L. hamatus* and *L. plomleyi*.

The upright portion of the femoral process “L” is doubly forked on the left gonopod of the paratype male (Fig. 3D). There is no second bifurcation on the right gonopod of the paratype, or on the femoral processes of the holotype, so the double forking appears to be a developmental abnormality. (The third, non-type male is missing its femoral processes.)

The holotype and paratypes were collected by Tasmanian land snail specialist Kevin Bonham in company with the author. We searched the group of rocks shown in Fig. 1C and similar nearby habitats for more than an hour but found no more *L. nivalis* sp. n., although we saw scattered specimens of the common northeast Tasmanian dalodesmids *L. adrianae* and *Tasmaniosoma clarksonorum* Mesibov, 2010. My collecting in November 2017 was even less successful, yielding only one presumed juvenile of *L. nivalis* sp. n., despite my searching a larger area of apparently suitable shrub habitat for several hours. *L. nivalis* sp. n. may be naturally scarce in its alpine habitat.

The types were collected live and transported from the field in a collecting jar filled with peaty material. As often happens when dalodesmids are live-collected, the female and one of the males (the holotype) mated in the jar and were still *in copula* when killed by freezing several hours later. In Fig. 2C, white amorphous material (spermatic fluid?) can be seen adhering to the gonopod telopodites of the holotype.

## Supplementary Material

XML Treatment for Lissodesmus

XML Treatment for Lissodesmus nivalis
